# Use of effective contraception following provision of the progestogen-only pill for women presenting to community pharmacies for emergency contraception (Bridge-It): a pragmatic cluster-randomised crossover trial

**DOI:** 10.1016/S0140-6736(20)31785-2

**Published:** 2020-11-14

**Authors:** Sharon T Cameron, Anna Glasier, Lisa McDaid, Andrew Radley, Paula Baraitser, Judith Stephenson, Richard Gilson, Claire Battison, Kathleen Cowle, Mark Forrest, Beatriz Goulao, Anne Johnstone, Alessandra Morelli, Susan Patterson, Alison McDonald, Thenmalar Vadiveloo, John Norrie

**Affiliations:** a Department of Obstetrics and Gynaecology, University of Edinburgh, Edinburgh, UK; b Edinburgh Clinical Trials Unit, Usher Institute, University of Edinburgh, Edinburgh, UK; c Chalmers Sexual and Reproductive Health, NHS Lothian, Edinburgh, UK; d Institute for Social Science Research, The University of Queensland, Brisbane, QLD, Australia; e MRC/CSO Social and Public Health Sciences Unit, University of Glasgow, Glasgow, UK; f Directorate of Public Health, NHS Tayside, Dundee, UK; g Division of Cardiovascular Medicines and Diabetes, Ninewells Hospital and Medical School, Dundee, UK; h Department of Sexual Health, King's College Hospital NHS Foundation Trust, London, UK; i Elizabeth Garrett Anderson Institute for Women's Health, University College London, London, UK; j Institute for Global Health, University College London, London, UK; k Boots UK, Edinburgh, UK; l Health Services Research Unit, University of Aberdeen, Aberdeen, UK

## Abstract

**Background:**

Unless women start effective contraception after oral emergency contraception, they remain at risk of unintended pregnancy. Most women in the UK obtain emergency contraception from community pharmacies. We hypothesised that pharmacist provision of the progestogen-only pill as a bridging interim method of contraception with emergency contraception plus an invitation to a sexual and reproductive health clinic, in which all methods of contraception are available, would result in increased subsequent use of effective contraception.

**Methods:**

We did a pragmatic cluster-randomised crossover trial in 29 UK pharmacies among women receiving levonorgestrel emergency contraception. Women aged 16 years or older, not already using hormonal contraception, not on medication that could interfere with the progestogen-only pill, and willing to give contact details for follow-up were invited to participate. In the intervention group, women received a 3-month supply of the progestogen-only pill (75 μg desogestrel) plus a rapid access card to a participating sexual and reproductive health clinic. In the control group, pharmacists advised women to attend their usual contraceptive provider. The order in which each pharmacy provided the intervention or control was randomly assigned using a computer software algorithm. The primary outcome was the use of effective contraception (hormonal or intrauterine) at 4 months. This study is registered, ISRCTN70616901 (complete).

**Findings:**

Between Dec 19, 2017, and June 26, 2019, 636 women were recruited to the intervention group (316 [49·6%], mean age 22·7 years [SD 5·7]) or the control group (320 [50·3%], 22·6 years [5·1]). Three women (one in the intervention group and two in the control group) were excluded after randomisation. 4-month follow-up data were available for 406 (64%) participants, 25 were lost to follow-up, and two participants no longer wanted to participate in the study. The proportion of women using effective contraception was 20·1% greater (95% CI 5·2–35·0) in the intervention group (mean 58·4%, 48·6–68·2), than in the control group (mean 40·5%, 29·7–51·3 [adjusted for recruitment period, treatment group, and centre]; p=0·011).The difference remained significant after adjusting for age, current sexual relationship, and history of effective contraception use, and was robust to the effect of missing data (assuming missingness at random). No serious adverse events occurred.

**Interpretation:**

Provision of a supply of the progestogen-only pill with emergency contraception from a community pharmacist, along with an invitation to a sexual and reproductive health clinic, results in a clinically meaningful increase in subsequent use of effective contraception. Widely implemented, this practice could prevent unintended pregnancies after use of emergency contraception.

**Funding:**

National Institute for Health Research (Health Technology Assessment Programme project 15/113/01).

## Introduction

Emergency contraception prevents unintended pregnancy after unprotected sex or contraceptive failure,^1^ but unless women start an effective method of ongoing contraception after oral emergency contraception, they remain at risk of unintended pregnancy. Women who have unprotected sex after receiving emergency contraception (in the same cycle) are up to three-times more likely to conceive than women who do not,^1^, ^2^, ^3^ and without contraception these women remain at risk of pregnancy in subsequent cycles. Current UK and US guidelines recommend initiating regular hormonal contraception immediately after emergency contraception (known as quick-starting).^4^, ^5^

In the UK, as in many other countries, most women obtain emergency contraception from community pharmacies without a prescription.^6^ However, for ongoing contraception, pharmacists can generally only provide barrier methods which have high failure rates.^7^ With the exception of a few areas in the UK in which local arrangements have been made for a finite supply of oral contraceptives from the pharmacist,^8^ most women need to attend a contraceptive provider such as their general practitioner or a sexual and reproductive health clinic, which takes time and women might lose motivation to seek contraception and the risk for unintended pregnancy increases.

**Research in context**

**Evidence before this study**

We searched PubMed for articles in English published from March 1, 2000, to March 30, 2020, using the search terms “bridging” OR “bridge” AND “emergency contracept”. We found 12 articles of which only two were relevant. One article was a cluster-randomised pilot study, done by the authors of this study, of 11 pharmacies in Edinburgh (UK) supplying 1 month of the progestogen-only pill plus emergency contraception, or an offer of rapid access to a local sexual and reproductive health clinic (upon presentation of a card to the clinic) where contraception would be supplied, or emergency contraception alone (control). In both intervention groups a significantly higher proportion of women (more than double) were using regular effective contraception after 6–8 weeks compared with the group receiving emergency contraception alone. The other article was a cluster-randomised trial in Jamaica and offered women seeking emergency contraception a discount voucher for a limited period (voucher had to be used within 2·5 to 4 months of recruitment) on the subsequent cost of contraceptive pills. This study reported no effect of the intervention on contraceptive use. The scarcity of data on this topic underlined the need for a high quality, adequately powered, randomised trial.

**Added value of this study**

Our study showed that pharmacy provision of a 3-month supply of progestogen-only pill and the offer of rapid access to a sexual and reproductive health clinic along with emergency contraception was associated with a 20% increase in use of effective contraception 4 months later (1 month after progestogen-only pill had run out) compared with provision of emergency contraception alone.

**Implications of all the available evidence**

Most women who use emergency contraception remain at risk of unintended pregnancy unless they start an effective method of contraception. The progestogen-only pill is a safe contraceptive with few contraindications, low cost, and high suitability for provision from the community pharmacy. An increase of 20% in effective contraceptive uptake was large and clinically significant. Offering a 3-month supply of progestogen-only pill and rapid access to a contraceptive service with emergency contraception might prevent many more unintended pregnancies if this practice became standard practice in UK pharmacies.

In 2012, we did a pilot study among 168 women presenting to 11 community pharmacies in Edinburgh (UK) for levonorgestrel-containing emergency contraception. Pharmacies offered a 1-month supply of a progestogen-only pill, or an invitation for rapid access to a sexual and reproductive health clinic for advice and provision of ongoing contraception, or standard advice about starting contraception after emergency contraception.^9^ When participants were followed up 6–8 weeks later, the proportion of women using effective contraception was significantly greater in both the progestogen-only pill group (22 [56%] of 39 women; p=0·001) and the rapid access group (13 [52%] of 25; p=0·006) compared with standard care (5 [16%] of 31).

Here, we present the findings of the full trial designed to support the findings of the pilot study. We used a composite intervention of a bridging supply of the progestogen-only pill and the offer of rapid access to a participating sexual and reproductive health clinic. This approach combined temporary contraception (giving women time to get an appointment with their usual contraceptive provider) with facilitated access to a specialist contraceptive service in which all methods of contraception, including the most effective methods of long-acting reversible contraception (LARC), were available. The aim of this study was to determine whether the composite intervention resulted in increased use of subsequent effective contraception (hormonal or intrauterine) compared with provision of emergency contraception alone.

## Methods

### Study design and participants

Bridge-It was a cluster-randomised cohort crossover trial including 29 pharmacies in three UK regions: London (south and central), Lothian (Edinburgh and region), and Tayside (Dundee and region).^10^ The order in which each pharmacy provided the intervention or control was randomly assigned, with an intervening period of at least 2 weeks (during which recruitment was halted). Each pharmacy recruited women into the intervention and the control cohorts. We chose cluster design because the pilot study^9^ showed that randomisation of individual participants (rather than pharmacies), would not recruit enough participants. The crossover design was chosen for efficiency so that each cluster was acting as its own control and fewer pharmacies were required. The washout period (planned duration of at least 2 weeks) between recruitment periods minimised the effect of the preceding intervention or control.^10^ Study pharmacies were chosen on the basis of their geographical position (within 5 miles of the sexual and reproductive health study clinics), the volume of emergency contraception dispensed (more than 30 each month), and the presence of a private interview area.

Participants were women receiving emergency contraception from a study pharmacy. The emergency contraception used in the study was levonorgestrel, given in the clinically indicated dose (1·5 mg or 3 mg) according to bodyweight.^4^ In the intervention group, women received three packets of the progestogen-only pill containing 28 tablets (75 μg desogestrel per day) at no cost. Locally approved Patient Group Directions (strict criteria to permit provision of specified medicines by non-prescribers) allowed pharmacists to dispense the progestogen-only pill to participants. Women aged 16 years or older, not already using hormonal contraception, not on medication that could interfere with the progestogen-only pill, and willing to give contact details for follow-up were invited to participate. Full inclusion and exclusion criteria are described in the appendix (p 1).^11^ Pre-study training for pharmacists was delivered using the study protocol and Patient Group Directions for the progestogen-only pill, which included information about medical contraindications, potential drug interactions, and missed pill guidance. Pharmacists were instructed to advise women to start the progestogen-only pill the day after emergency contraception. As part of the intervention, women also received a rapid access card that upon presentation to the local participating sexual and reproductive health clinic enabled them to discuss and obtain alternative effective contraception, including LARC. The card provided information about the location and opening hours of the sexual and reproductive health clinic, which provided consultations and all methods of contraception at no cost, as is the norm in the UK National Health Service (NHS). In the control group, women received standard care (emergency contraception and advice about ongoing contraception). Mystery shopper visits were done just before control group recruitment started to document the content of standard care consultations on emergency contraception and these data have been published.^12^

A multi-method process evaluation was also conducted to assess implementation, mechanisms of change, and context,^10^ and included qualitative interviews with participants, pharmacists, and staff at sexual and reproductive health clinics (findings will be reported separately).

Pharmacists assessed women's eligibility for the study (appendix p 2), invited them to participate, and obtained written informed consent for the study (including access to sexual and reproductive health records and data linkage with the national abortion registries). Pharmacists also provided a patient information sheet. Participants completed a baseline questionnaire that included demographic details, reproductive history, and previous methods of contraception used including emergency contraception. To maximise retention in the study, participants received an incentive voucher of £10 at recruitment.^13^ Pharmacies received £30 per participant enrolled onto the study to cover costs of the time spent recruiting participants. Ethical approval was obtained from the South East Scotland Research Ethics Committee in June, 2017. Approvals were also obtained from NHS Research Scotland and the Health Research Authority in England. The full study protocol is available in the paper published in 2019, by Cameron and colleagues.^10^

### Randomisation and masking

Eligible participants who met the criteria were enrolled to receive the progestogen-only pill plus a rapid access card to a sexual and reproductive health clinic (intervention group) or received advice from a pharmacist to attend their usual contraceptive provider (control group). The order in which pharmacies delivered the control or intervention was randomised for each pharmacy, and generated using a computer software algorithm that randomly allocated permuted blocks of size 2, 4, and 6; blocking was used to ensure a balanced order. The randomisation file was prepared by a study statistician at the Centre for Healthcare Randomised Trials (University of Aberdeen, UK), using SAS version 6.4. The study statistician was masked to the outcome assessment, but women and research nurses were not masked. Pharmacists were informed of the randomised allocation by the study trial manager at the Edinburgh Clinical Trials Unit.

### Procedures

Participants in both groups had a single follow-up at 4 months through a telephone interview with a research nurse or a self-administered online questionnaire. The follow-up method was chosen according to participants' preference. Participants who did not respond to three attempts to contact them by telephone or email were considered lost to follow-up. In addition to contraceptive use, women were asked about their interaction with the pharmacist and use of the rapid access card (intervention group only). Participants reporting pregnancy also completed the London Measure of Unplanned Pregnancy questionnaire—validated to measure the intendedness of pregnancy.^14^, ^15^ Only serious adverse events were recorded from start of recruitment to the 4-month follow-up. Other adverse events were not recorded because both the emergency contraception and progestogen-only pill are approved medicines used for licensed indications. The participating sexual and reproductive health clinics also searched records for data on whether, and for what reason, participants in both groups had used their service during the 4 months following recruitment.

### Outcomes

The primary outcome was self-reported use of effective contraception (hormonal or intrauterine) at 4 months. Secondary outcomes were incidence of abortion in the 12 months following recruitment and an economic evaluation of the intervention. Both secondary outcomes will be reported separately. A post-hoc analysis was also done, adjusting (in addition to the prespecified baseline covariates) for ethnicity and having given birth.

### Statistical analysis

The original power calculation was based on two coprimary outcomes; abortion rates at 12 months after recruitment and effective contraception use at 4 months follow-up. The abortion outcome at 12 months required over 2000 women to be enrolled in more than 26 pharmacies to have 90% power at 2·5% level of significance to detect a relative reduction in the abortion rate of around 50%—eg, from 8% to 4%. Recruiting this large number of women was not feasible within the available timeframe and resources. Without seeing the unblinded data, the independent oversight committees (Trial Steering Committee and Data Monitoring Committee) and the funder (National Institute for Health Research [Health Technology Assessment Programme]) agreed to repower the study on a single coprimary outcome (effective contraceptive use at 4 months) to have 90% power at 5% level of significance to show an increase in effective contraception use from around 30% to 45% (absolute change 15%, relative increase 50%). For this outcome, the study required between 626 and 737 women, depending on the intraclass correlations (within cluster-periods and between cluster-periods), which could not be observed within the study at the time this change was made.^16^ This calculation assumed that 25% of women would be missing the outcome (lost to follow-up) at 4 months and that 25 pharmacies would be participating.

The baseline characteristics and 4-month follow-up data were summarised using mean (SD) or median (IQR) for continuous variables. Discrete variables were summarised with numbers and percentages. Analyses were done on the intention to treat principle. For the primary outcome, the percentage of women reporting effective contraception use in each cluster-period was analysed using linear regression adjusting for period, treatment group, and centre.^17^ The published study protocol^10^ specified a hierarchical mixed effect, logistic regression on individual data for the analysis of effective contraception use at 4 months. We revised this analysis in the final Statistical Analysis Plan (accepted Nov 5, 2019, before any unblinded data had been seen) to use a linear model on the unweighted proportion (expressed as a percentage), with the same primary outcome at site level, following methodological guidance from Morgan and colleagues,^17^ and we used the hierarchical mixed effects logistic regression as a sensitivity type analysis. Although the linear model on percentages at site level makes less use of the available information, it can be more robust, with fewer assumptions, and expresses the treatment effect as a percentage difference in proportion rather than an odds ratio.

Prespecified baseline covariates (mean age, percentage of participants who were in an ongoing sexual relationship, and percentage of participants who had used effective contraception methods previously) were included in an additional analysis. A two-sided p value of <0·05 was taken as statistically significant. For the primary outcome, prespecified subgroup analysis was done for LARC use according to the classification by the UK National Institute for Health and Care Excellence (intrauterine contraception, implant, injectable)^18^ with a more stringent definition of statistical significance (two-sided 1% significance level with 99% CI). Sensitivities of treatment effect estimate for missing outcome data was analysed using multiple imputation. All analyses were done with STATA (version 16). An independent data monitoring committee oversaw the study. The trial registry number is ISRCTN70616901.

### Role of the funding source

The funder of the study had no role in study design, data collection, data analysis, data interpretation, or writing of the report. The corresponding author had full access to all the data in the study and had final responsibility for the decision to submit for publication.

## Results

**Figure 1:**
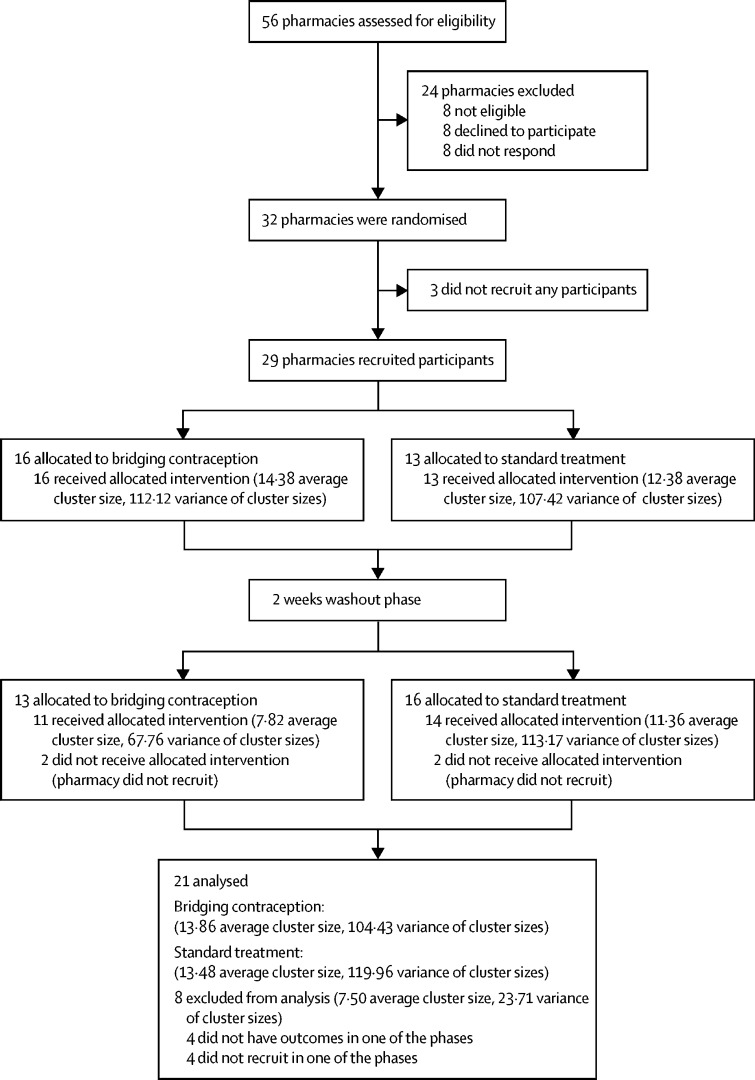
Trial profile

**Figure 2:**
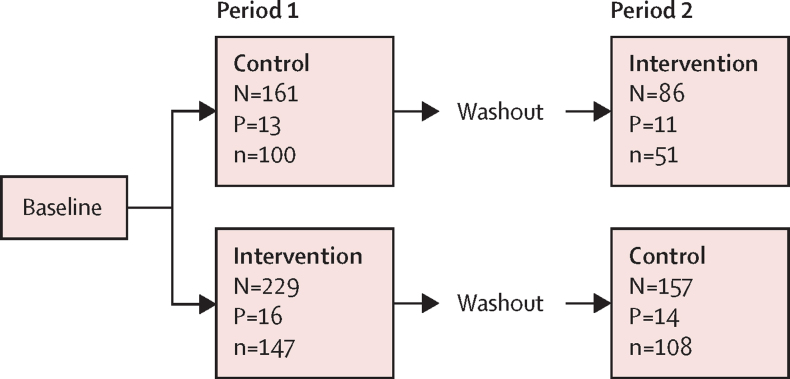
Flowchart of participants N is the number of women recruited, P is the number of pharmacies, and n is the number of women providing follow-up at 4 months.

Of 56 pharmacies approached, 32 agreed to take part and between Dec 19, 2017, and June 26, 2019, 29 pharmacies were recruited (14 pharmacies in London, 12 in Edinburgh, and three in Tayside; figure 1). The change to include only a single primary outcome took place in January, 2019. A total of 1252 participants were screened; 762 were eligible, and 636 participants gave consent and were enrolled. Three women (one in the intervention group and two in the control group) were excluded after randomisation. The trial profile of participants is shown in figure 2. The median number of participants recruited in each pharmacy was 17 (range 2–73). The number of participants recruited in each period of the study for each pharmacy ranged from two to 35 in phase 1 of recruitment and from 0 to 38 in period 2 of recruitment. Reasons for ineligibility are shown in the appendix (p 2).

Baseline characteristics of participants by randomised group and study period are summarised in table 1. The mean age of participants was 22·7 years (SD 5·7) in the intervention group and 22·6 years (5·1) in the control group. Follow-up data at 4 months were available for 198 (63%) of 315 women in the intervention group and 208 (65%) of 318 in the control group. Follow-up rates did not differ statistically between study groups or between those recruited in period 1 or period 2. There were no differences between responders and non-responders in any baseline characteristics (appendix p 3). No serious adverse events were reported in either group.

**Table 1:** Baseline characteristics

	**Intervention group, period 1 (N=229)**	**Intervention group, period 2 (N=86)**	**Control group, period 1 (N=161)**	**Control group, period 2 (N=157)**
**Age, years**				
Mean	23·2 (6·0)	21·4 (4·8)	22·2 (4·4)	22·9 (5·8)
**Methods of contraception used**				
Combined hormonal contraceptive (pill, patch, or ring)	114 (49·8%)	41 (47·7%)	99 (61·5%)	94 (59·9%)
Progestogen-only pill	39 (17·0%)	19 (22·1%)	35 (21·7%)	32 (20·4%)
Male condom	189 (82·5%)	66 (76·7%)	117 (72·7%)	135 (86·0%)
Progestogen-only injectable	14 (6·1%)	6 (7·0%)	10 (6·2%)	18 (11·5%)
Progestogen-only implant	29 (12·7%)	10 (11·6%)	23 (14·3%)	19 (12·1%)
Copper-bearing intrauterine device	6 (2·6%)	0	4 (2·5%)	4 (2·5%)
Levonorgestrel-releasing intrauterine system	0	1 (1·2%)	4 (2·5%)	2 (1·3%)
Withdrawal method	65 (28·4%)	28 (32·6%)	52 (32·3%)	67 (42·7%)
Other methods*	10 (4·4%)	6 (7·0%)	7 (4·3%)	9 (5·7%)
Never used any method	8 (3·5%)	4 (4·7%)	10 (6·2%)	2 (1·3%)
**Sexual and reproductive history**				
Previous birth	30 (13·1%)	5 (5·8%)	7 (4·3%)	13 (8·3%)
Previous termination	38 (16·6%)	6 (7·0%)	22 (13·7%)	27 (17·2%)
Previous miscarriage	17 (7·4%)	5 (5·8%)	10 (6·2%)	6 (3·8%)
Current sexual relationship	176 (76·9%)	55 (64·0%)	104 (64·6%)	111 (70·7%)
First time use of emergency contraception	52 (22·7%)	22 (25·6%)	28 (17·4%)	32 (20·4%)
**Number of times emergency contraception used in past 12 months**				
Mean	1·4 (1·4)	1·5 (1·5)	1·5 (1·2)	1·7 (2·0)
Median	1·0 (0·0–2·0)	1·0 (1·0–2·0)	1·0 (1·0–2·0)	1·0 (1·0–2·0)
Minimum, maximum	0·0–8·0	0·0–9·0	0·0–6·0	0·0–20·0
**Ethnic background**				
White	157 (68·6%)	60 (69·8%)	98 (60·9%)	114 (72·6%)
Asian or Asian British	21 (9·2%)	6 (7·0%)	8 (5·0%)	21 (13·4%)
Black or Black British	29 (12·7%)	12 (14·0%)	36 (22·4%)	15 (9·6%)
Mixed or other	19 (8·3%)	6 (7·0%)	17 (10·6%)	6 (3·8%)
Not specified	3 (1·3%)	2 (2·3%)	2 (1·2%)	1 (0·6%)

Data are mean (SD), N (%), or median (25th, 75th percentile). N is the number of women recruited. The proportion of women with previous history of ectopic pregnancy was less than 1% in all groups.

* Other methods of protection were female condom, cap or diaphragm, vasectomy, fertility awareness, and emergency contraception.

The proportion of women reporting use of effective contraception (hormonal and intrauterine; table 2) after adjustment was 20·1% higher (95% CI 5·2–35·0; p=0·011) in the intervention group (58·4%, SD 21·6) than in the control group (40·5%, 23·8). Effective contraception use remained significantly higher in the intervention group even when adjusted for recruitment period, treatment group, study centre, age, current sexual relationship, previous use of effective contraception, ethnicity, and previously giving birth, and was robust to the effect of missing data (table 3) shown by using a multiple imputation approach under an assumption of missing at random.

**Table 2:** Primary analysis for cluster-level models

	**Intervention group**	**Control group**	Estimate*(95% CI)	**p value**
Primary outcome†	21; 58·4% (21·6)	21; 40·5% (23·8)	20·1% (5·2–35·0)	0·011
Primary outcome adjusted‡	21; 58·4% (21·6)	21; 40·5% (23·8)	14·5% (0·9–28·2)	0·038
Primary outcome adjusted with additional covariates§	21; 58·4% (21·6)	21; 40·5% (23·8)	18·5% (1·4–35·6)	0·036

Data are N; mean (SD), unless otherwise specified. N is the number of pharmacies. Outcome is the percentage of effective contraception uptake at 4 months follow-up.

* The estimated treatment effect (percentage difference between groups) in proportion with the outcome.

† Adjusted for phase, treatment group, and centre.

‡ Adjusted for phase, treatment group, centre, mean age, percentage of participants in a current sexual relationship, and percentage of participants who had used effective contraception previously.

§ Post-hoc analysis adjusted in addition to the prespecified baseline covariates for ethnicity and having given birth previously.

**Table 3:** Sensitivity analysis

	**Intervention group**	**Control group**	Estimate*(95% CI)	**p value**
**Cluster-level models**†				
Omit cluster with <3 responses	14; 60·2% (19·7)	14; 42·9% (13·8)	15·2% (2·3 to 28·1)	0·026
Omit cluster with >30% individuals missing data (excluding centre)	5; 58·5% (14·4)	5; 44·1% (6·6)	14·7% (1·1 to 28·2)	0·040
Including percentage missing and number of responders	21; 58·4% (21·6)	21; 40·5% (23·8)	15·3% (−0·1 to 30·6)	0·051
Including percentage missing	21; 58·4% (21·6)	21; 40·5% (23·8)	15·0% (0·3 to 29·7)	0·046
Including number of responders	21; 58·4% (21·6)	21; 40·5% (23·8)	15·0% (0·6 to 29·4)	0·042
Multiple imputation	..	..	15·0% (−2·0 to 32·0)	0·076
**Hierarchical models**‡				
Fixed effects for cluster (n=397)	112/198 (56·6%)	85/208 (40·9%)	1·93%§ (1·21 to 3·09)	0·0058

Data are N; mean (SD), or n/N (%), unless otherwise specified. N is the number of pharmacies.

* The estimated treatment effect (percentage difference between groups) in proportion with the outcome.

† Outcome is the percentage of effective contraception uptake and analyses were adjusted for phase, treatment group, centre, mean age, percentage of participants who were in a current sexual relationship, and percentage of participants who had used effective contraception previously.

‡ Outcome is the uptake of effective contraception (binary) and was adjusted for phase, treatment group, and centre.

§ Odds ratio.

The methods of contraception used by participants at 4 months after emergency contraception are shown in the appendix (p 4). The most commonly used method was the progestogen-only pill in the intervention group and combined hormonal contraception in the control group. None of the women were sterilised or relying on a vasectomy. LARC use was not significantly different between the intervention group (13 [7%] of 198) and control group (23 [11%] of 208, 95% CI −10·04% to 1·05%; p=0·112). The most common reason given by women for not using an effective contraceptive method at 4 months was that they were not currently sexually active (appendix p 5). Significantly fewer women in the intervention group (20 [10%]) compared with the control group (37 [18%], −15·38% to −1·48%; p=0·018) had used emergency contraception again since recruitment (appendix p 5). Significantly more women in the intervention group (194 [98%] *vs* 157 [76%]) reported that the pharmacist had provided information about starting ongoing contraception and where to access it (appendix p 6). Of 198 respondents in the intervention group, 158 (80%) reported using some of the supplied progestogen-only pill. Data on when these participants started the progestogen-only pill, number of pill packets used, and reasons for non-use or discontinuation are shown in the appendix (p 7).

Data from the 4-month follow-up interview on use of the rapid access card, contraception supplied, and experience at the sexual and reproductive health clinic are shown in the appendix (p 8). Most respondents in the intervention group (137 [69%] of 198) could recall receiving the rapid access card, but only 31 (19%) attended the local sexual and reproductive health clinic preferring to obtain contraceptive supplies from their general practitioner. Only 17 (55%) of those 31 remembered to take the rapid access card with them. Of 31 (16%) women in the intervention group who attended the sexual and reproductive health clinic, only two used the rapid access card within 1 month of recruitment. Five of 31 rapid access card users received a LARC method (appendix p 8).

Data obtained from the sexual and reproductive health clinic showed that a similar proportion of participants in both groups attended the clinics within 4 months of recruitment (52 [17%] of 305 in the intervention group *vs* 43 [14%] of 309 in the control group, 95% CI −2·60% to 8·87%; p=0·284). Contraception was supplied to 41 (43%) of 95 clinic attendees and the methods of contraception are shown in the appendix (p 9). The most common method supplied to women in the intervention group was the progestogen-only pill. A total of 75 women attended the sexual and reproductive health clinic and provided data at the 4-month follow-up interview. In 34 of 75 cases, the sexual and reproductive health clinic provided a contraceptive method. Of these 34, 30 (88%) women reported using the same method of contraception at 4 months as the method provided by the sexual and reproductive health clinic.

A total of 19 (4·7%) respondents (nine in the intervention and ten in the control group) reported that they had been pregnant (17) or were currently pregnant (two) since recruitment. In ten of 19 cases the pregnancy ended in abortion, six women miscarried, and the outcome of one pregnancy was unknown. Based on scores from the London Measure of Unplanned Pregnancy questionnaire,^15^, ^16^ seven of nine pregnancies in the intervention group and eight of ten in the control group were clearly unintended.

## Discussion

The Bridge-It study showed that this simple pharmacist-delivered intervention, in which a bridging supply of the progestogen-only pill was given to women requesting emergency contraception along with the offer of rapid access to a sexual and reproductive health clinic, resulted in a significantly higher proportion of women using an effective method of contraception 4 months later compared with when emergency contraception was supplied alone. The difference in uptake of effective contraception between groups was large and likely to be clinically meaningful. Additionally, significantly fewer women in the intervention group used emergency contraception again, during these 4 months.

Aside from our pilot study,^9^ to our knowledge, this is the only published study designed to encourage uptake of effective contraception after emergency contraception offered women in Jamaica a time-limited discount for the cost of contraceptive pills, but the intervention had no effect on subsequent contraceptive use.^19^ Another intervention that has shown a clinical effect on the uptake of effective contraception is the CHOICE project from the USA.^20^ In this study,^20^ over 9000 women requiring regular contraception were counselled about LARC and provided with their chosen method free of charge in a setting in which contraception is usually not free (and LARC is expensive). The CHOICE project reported that LARC uptake was 67% compared with the national rate of 3% in the USA. By contrast, in the Bridge-it study, we assessed an intervention in a pharmacy setting at request for emergency contraception. This intervention was simpler to deliver and more affordable.

The Bridge-it study was a well-designed trial with an appropriate sample size to show the expected effect with sufficient power. This pragmatic trial had few inclusion and exclusion criteria,^21^ and included a mix of large chain and small independent pharmacies that dispensed emergency contraception at a high volume, so the results are probably generalisable to UK pharmacies in which most emergency contraception is provided. The study design was shaped by learning from the pilot study^9^ and this larger trial confirmed the pilot study findings in a more generalised setting. The findings were robust and remained highly statistically significant after adjustment for a range of relevant factors and for missing data. Women who participated in this study had similar characteristics to emergency contraception users nationally, suggesting that they are representative of women in the UK seeking emergency contraception to avoid an unintended pregnancy.^9^, ^22^

One of the challenges when conducting this study was that pharmacists are typically unfamiliar with participating in research.^23^ Dispensing emergency contraception can be time-intensive and, thus, asking pharmacists to recruit participants, provide further information, and complete additional paperwork as part of a research study was an added burden. Consequently, recruitment took longer than anticipated and there were large differences between pharmacies in the number of women recruited and within pharmacies in the number recruited in each period.

As expected, there was a substantial drop-out rate for the self-reported primary outcome of effective contraception use at 4 months. We had estimated this loss to be at 25% and in the trial it was 35%. However, there was no differential loss to follow-up either between treatment groups or between recruitment periods. We checked the robustness of our findings to the effect of these missing data using a multiple imputation approach under an assumption of missing at random and the findings were confirmed. In addition, the characteristics of participants who did and did not provide follow-up data were similar. Although from a methodological perspective it would have been ideal for every pharmacy to have contributed an equal number of participants in both periods, for a pragmatic trial and to make the findings relevant to routine practice, we enlisted a range of pharmacy sizes with differing numbers of pharmacists (from single-handed to multiple pharmacist stores). The cohort design meant that different groups of women would participate in the two periods. All these factors contributed to varying recruitment rates across pharmacies and between periods, resulting in different lengths of required recruitment. Although this design was more difficult to manage, it produced more robust and generalisable findings than randomisation at an individual participant level.

Pregnancy was not the primary endpoint in this study. Although we still plan to record linkage with abortion statistics 12 months after emergency contraception use (number of births anticipated to be few and the relevant time frame for follow-up anticipated to be longer), this study was underpowered for this comparison. Use of effective contraception should prevent unintended pregnancy and is used as a surrogate measure in contraceptive research studies. The effectiveness of contraception is dependent on continuity and correct use; however, discontinuation^24^ and incorrect use^25^ are common. Longer follow-up was not possible in this study but would have provided data on continuation of the chosen method. Contraceptive use was self-reported and published evidence shows that women's self-reporting of the contraceptive method is reasonably reliable.^26^ Enthusiasm among study pharmacists could have resulted in a higher than usual proportion of women in the control group receiving advice about the importance of ongoing contraception than under non-study conditions. Notably, three-quarters of women in the control group of the Bridge-it trial received advice about contraception compared with a half of women in the mystery shopper study designed to characterise standard care.^12^

An important factor for effective contraceptive use in the intervention group was continued use of the progestogen-only pill, as more women in the intervention group than in the control group requested further supplies of the progestogen-only pill from sexual and reproductive health clinics and their general practitioner. In the pilot study and this trial, the progestogen-only pill was the subsequent method of choice for a large proportion of women, even at sexual and reproductive health clinics with the full range of methods available.^9^ The progestogen-only pill has not been a common first choice among women in the UK; it is estimated to be used by only 5·6% of prescription contraceptive users,^27^ and is usually reserved for those with contraindications to the combined oral contraceptive pill.^28^ Perhaps, the progestogen-only pill is not widely offered to women by providers, but when given the option, women decide to continue use, as observed in this trial.

The intervention could also have served as a reminder to the pharmacist to discuss contraception, given that more women in the intervention group than in the control group received advice about ongoing contraception. In our earlier pilot study women were given only a 1-month supply of the progestogen-only pill.^9^ Our hypothesis for the pilot study was that around the time of seeking emergency contraception, women might be highly motivated to arrange a visit to their contraceptive provider to obtain ongoing contraception and receiving a 1-month supply of the progestogen-only pill gave them sufficient time to arrange a visit. We were concerned that a 3-month supply of the progestogen-only pill would be too much, as motivation to seek ongoing contraception might dissipate with time, but findings from the Bridge-it study showed otherwise.

The rapid access card to a sexual and reproductive health clinic was used by fewer than one in five women (compared with 32% in the earlier pilot study),^9^ and only two women (1% of the total intervention) used this access card rapidly (within 1 month). Also, the rapid access card did not result in more women using the sexual and reproductive health clinic compared with the control group. The rapid access component was intended to facilitate access to LARC but only 16% of rapid access card users received LARC. Although we cannot conclude that the rapid access does not make a meaningful contribution to accessing effective contraception for some, the findings suggest that this component might be less important than the bridging progestogen-only pill intervention.

Despite free contraceptive services in the UK, unintended pregnancy remains a public health problem.^29^ In 2018 there were 200 608 induced abortions for women in England and Wales and 13 286 in Scotland.^30^, ^31^ In England and Wales this number represented an increase of 4% since 2017 and the highest number recorded, whereas the figures for Scotland represent a 10-year high. What would happen if offering 3-months' supply of the progestogen-only pill with emergency contraception became standard practice throughout the UK? An increase of 20% in contraceptive uptake is large and clinically significant. Additionally, pharmacy provision of an oral contraceptive is inexpensive. A 3-month supply of the desogestrel progestogen-only pill costs the NHS £2·97 and the reimbursed cost of the pharmacist's time is likely to be around £30.^32^, ^33^ If this approach was routinely implemented as a service without the need to participate in a research study, many more women might accept the offer of the progestogen-only pill with emergency contraception. A survey conducted in 2013, involving over 100 women seeking emergency contraception from pharmacies in Edinburgh, showed that two-thirds of women welcomed the prospect of receiving a bridging supply of the progestogen-only pill along with emergency contraception.^22^ This bridging method has also received support from members of the Faculty of Sexual and Reproductive Healthcare UK, gathered from surveys.^22^, ^34^ The progestogen-only pill is safe and has few contraindications to use.^28^ In addition, current threats of the pandemic to health-care delivery show the importance of developing alternative methods for supplying contraception.

In addition to increasing uptake of effective contraception, it is hoped that implementing bridging widely could reduce the rates of repeat use of emergency contraception, particularly among those who are most at risk of an unintended pregnancy. This point is particularly important, as findings from the British National Survey of Sexual Attitudes and Lifestyles (NATSAL-3),^29^ done between 2010 and 2012, highlight that emergency contraception users represent a group of women at greater risk of unintended pregnancy, and that use of emergency contraception has risen in this group.^6^ Thus, it is unsurprising that the pregnancy rate at 4·7% shown in the Bridge-it study in the first 4 months of follow-up seems considerable. NATSAL-3 also found that use of emergency contraception had increased among single women, those living in less affluent areas, and those obtaining emergency contraception from retail rather than health-care sources.^6^ This finding highlights the potential for providing bridging contraception within retail settings, such as community pharmacies, to reduce repeat emergency contraception use and unintended pregnancies in this group.^35^ Importantly, the mean age of women participating in the Bridge-it study was 22 years, highlighting that the intervention reached key age groups; abortion rates in the UK are highest among women aged 20–24 years^30^, ^31^ and use of emergency contraception highest among women aged 16–24 years.^6^ Thus, if the Bridge-it intervention were implemented widely as a service, the use of effective contraception might increase in the relevant age group and among those at greater risk of unintended pregnancy.

Some might argue that our intervention could undermine attempts to encourage use of LARC. However, there were no significant differences between the two groups in this study in the uptake of LARC after emergency contraception use. It was disappointing that few women in the intervention group used the rapid access card which gave priority access to sexual and reproductive health services offering LARC. Data from interviews with participants, pharmacists, and sexual and reproductive health clinic staff, done as part of the evaluation (to be published separately), will provide more understanding. However, there are many women who do not want to use LARC,^36^ and they might be more likely to present for emergency contraception. Modelling from the USA^37^ has shown that a greater proportion of unintended pregnancies at population level could be prevented if efforts focused on encouraging sexually active women who are not using an effective contraceptive method (eg, progestogen-only pill) rather than switching those already using an effective method to a more effective LARC method.

We chose to provide the progestogen-only pill as bridging contraception rather than the more commonly used combined oral contraceptive pill, because the progestogen-only pill has fewer contraindications and health risks.^38^ The progestogen-only pill (75 μg desogestrel per day) consistently inhibits ovulation in almost all users (by contrast with the first-generation progestogen-only pills), and can be missed for 12 h without jeopardising effectiveness,^28^ and so is likely to be as effective as the combined oral contraceptive. The progestogen-only pill might also be easier to use because it is taken every day without a break.

Our study involved levonorgestrel-containing emergency contraception, which was the most common emergency contraception used at the time,^39^ and is suitable for immediate start of hormonal contraception.^4^ However, another oral emergency contraception, ulipristal acetate, has been shown to be more effective and is now widely available from UK community pharmacies.^40^ Future rollout of this intervention does not need to be limited to levonorgestrel-containing emergency contraception and can be adapted for other methods. The progestogen-only pill could be provided alongside ulipristal acetate with the recommendation to wait 5 days before starting the progestogen-only pill, to avoid a possible negative effect of the contraception on the effectiveness of ulipristal acetate, given that ulipristal is a progesterone receptor modulator.^4^

The Bridge-it study provides robust evidence that provision of a bridging supply of the progestogen-only pill with emergency contraception from a community pharmacy, together with an invitation to a sexual and reproductive health clinic, result in a clinically important increase in continued effective contraception use compared with provision of emergency contraception alone. Widescale implementation of this simple and safe intervention is now indicated and should be expected to reduce the rates of unintended pregnancy after emergency contraception.

## Data sharing

All data requests should be submitted to STC (the chief investigator) for consideration. Access to the anonymised data might be granted following review by the chief investigator and Edinburgh Clinical Trials Unit.
