# Supplementary material for: Use of effective contraception following provision of the progestogen-only pill for women presenting to community pharmacies for emergency contraception (Bridge-It): a pragmatic cluster-randomised crossover trial
Source: Lancet. 2020 Nov 14;396(10262):1585–94. doi: 10.1016/S0140-6736(20)31785-2 (PMC7661838; doi:10.1016/S0140-6736(20)31785-2)
Supplement: Supplementary appendix [file mmc1.pdf]

THE LANCET

Supplementary appendix

This appendix formed part of the original submission and has been peer reviewed. We post it as supplied by the authors.

Supplement to: Cameron ST, Glasier A, McDaid L, et al. Use of effective contraception following provision of the progestogen-only pill for women presenting to community pharmacies for emergency contraception (Bridge-It): a pragmatic cluster-randomised crossover trial. *Lancet* 2020; **396:** 1585–94.

Supplemental Table 1. Inclusion and exclusion criteria and study outcomes

**Inclusion Criteria**<br>• Intake of EC (levonorgestrel)<br>• Able to give informed consent to participate in and to adhere to trial requirements<br>• Age >=16 years<br>• Willing to give contact details and be contacted at four months by phone / SMS/e-mail or post<br>• Willing to give identifying data sufficient to allow data linkage with NHS registries
**Exclusion Criteria**<br>• Contraindications to POP<br>• Taking medication that interacts with POP<br>• Already using hormonal contraception<br>• Requires interpreting services<br>• Pharmacist has concerns about non-consensual sex

POP= progestogen only pill

EC= emergency contraception

Supplemental Table 2. Known reasons for ineligibility of those screened

Reasons for ineligibility* (N=490)	n (%)
Does not require emergency contraception	93(19.0)
Does not have capacity to give informed consent	64(13.1)
<16 years old	10(2.0)
Not willing to give contact details and be contacted for follow up	264(53.9)
Not willing to give identifying data sufficient to allow data linkage with NHS registries	262(53.5)
Contraindication to the POP	15(3.1)
On medication that interacts with POP	5(1.0)
Already using a hormonal method of contraception	156(31.8)
Requires an interpreting service	10(2.0)
Pharmacist has concerns about non-consensual sex	2(0.4)

*women may appear in more than one category

POP= progestogen only pill

Supplemental Table 3. Characteristics of responders and non-responders at four months (baseline data collected at recruitment)

Baseline variables		Non-responders	Responders
		N=227	N=406
Age, mean (SD)		22.3(5.0)	22.8(5.7)
**Methods of contraception ever used**			
Combined hormonal contraceptive (pill/patch/ring)		118(52.0%)	230(56.7%)
Progestogen only pill		46(20.3%)	79(19.5%)
Male condom		157(69.2%)	350(86.2%)
Progestogen only injectable		19(8.4%)	29(7.1%)
Progestogen only implant		29(12.8%)	52(12.8%)
Cu-IUD		3(1.3%)	11(2.7%)
LNG-IUS		2(0.9%)	5(1.2%)
Female condom		0	3(0.7%)
Cap or Diaphragm		0	6(1.5%)
Vasectomy		1(0.4%)	3(0.7%)
Withdrawal method		62(27.3%)	150(36.9%)
Fertility based awareness method		7(3.1%)	13(3.2%)
Other method		1(0.4%)	2(0.5%)
Never Used Any Method		11(4.8%)	13(3.2%)
Previous birth	Yes	16(7.0%)	39(9.6%)
Previous abortion	Yes	36(15.9%)	57(14.0%)
Previous miscarriage	Yes	16(7.0%)	22(5.4%)
Current sexual relationship	Yes	154(67.8%)	292(71.9%)
First time ever use of EC	Yes	44(19.4%)	90(22.2%)
No. times used EC in the last 12 months	Mean (SD)	1.5,(1.2)	1.5,(1.7)
	Median (25th, 75th centile)	1.0;[1.0,2.0]	1.0;[1.0,2.0]
	Min, Max	(0.0,6.0)	(0.0,20.0)
Ethnic Background	White	134(59.0%)	295(72.7%)
	Asian or Asian British	25(11.0%)	31(7.6%)
	Black or Black British	42(18.5%)	50(12.3%)
	Mixed or Others	23(10.1%)	25(6.2%)
	Missing	3(1.3%)	5(1.2%)

Cu-IUD = copper bearing intrauterine device

LNG-IUS= levonorgestrel releasing intrauterine system

Supplemental Table 4. Contraceptive use at four-months follow up

Variables		Intervention N=198	Control N=208
Methods of contraception used now*	Combined hormonal contraceptive pill/patch/ring	28/198(14.1%)	47/208(22.6%)
	POP	71/198(35.9%)	15/208(7.2%)
	Male condom	31/198(15.7%)	63/208(30.3%)
	Progestogen only injectable	4/198(2.0%)	4/208(1.9%)
	Progestogen only implant	3/198(1.5%)	11/208(5.3%)
	Cu- IUD	3/198(1.5%)	4/208(1.9%)
	LNG-IUS	3/198(1.5%)	4/208(1.9%)
	Other method	0	1/208(0.5%)
	Not using any method	57/198(28.8%)	62/208(29.8%)
	LARC**	13/198(6.5%)	23/208 (11.1%)
When did the participant start using this contraceptive method	The same day that took the EC	18/141(12.8%)	14/146(9.6%)
	The day after took the EC	38/141(27.0%)	7/146(4.8%)
	With start of period after EC	13/141(9.2%)	23/146(15.8%)
	Other	48/141(34.0%)	62/146(42.5%)
	Missing	24/141(17.0%)	40/146(27.4%)
Where did the participant get the current methods of contraception from	GP Clinic	74/141(52.5%)	53/146(36.3%)
	At a SRH clinic	34/141(24.1%)	35/146(24.0%)
	Other	21/141(14.9%)	40/146(27.4%)

POP= progestogen only pill
EC= emergency contraception
Cu-IUD = copper bearing intrauterine device
LNG-IUS= levonorgestrel releasing intrauterine system
*Participants could use more than one method
**LARC includes Cu-IUD, LNG-IUS, progestogen only implant and injectable

Supplemental Table 5. Reasons for not using effective contraception at four months and further use of emergency contraception

		**Intervention N=57***	**Control N=62**
Reasons for not using effective contraception	Not currently sexually active	27/57(47.4%)	28/62(45.2%)
	Worried about side effects with contraception	12/57(21.1%)	21/62(33.9%)
	Due to medical reasons	1/57(1.8%)	2/62(3.2%)
	Not decided on method to be used	9/57(15.8%)	11/62(17.7%)
	Difficult to get appointment for GP or a SRH clinic	8/57(14.0%)	4/62(6.5%)
	Difficult to find time to get to GP or a SRH clinic	6/57(10.5%)	4/62(6.5%)
	Trying for baby	1/57(1.8%)	0
	Other	8/57(14.0%)	5/62(8.1%)
Further use of EC since entering study	Yes	20/198(10.1%)	37/208(17.8%)
How many times EC used since	Mean (SD)	1.6,(1.2);[N=19]	1.5,(0.8);[N=36]
	Median (25th centile, 75th centile)	1.0;[1.0,2.0]	1.0;[1.0,2.0]
	Min, Max	(1.0,5.0)	(1.0,5.0)

EC= emergency contraception
GP= general practitioner
SRH= sexual and reproductive health clinic (any)

*45 out of these 57 women in the intervention group (79%) used the POP supplied by the pharmacist

Supplemental Table 6. Provision of ongoing contraceptive advice by pharmacy as reported by participants at four-months follow-up.

		Intervention	Control	Effect size
		N=198	N=208	
Did the pharmacy provide participant with information about starting contraception	No	2(1.0%)	46(22.1%)	0.216(0.156,0.277);
	Yes	194(98.0%)	157(75.5%)	***p<0.001***
Did the pharmacy provide participant with information about where to get contraception	No	19(9.6%)	67(32.2%)	0.237(0.159,0.315);
	Yes	178(89.9%)	134(64.4%)	***p<0.001***

Supplemental Table 7: Use of progestogen only pill (POP) over four months by participants in intervention group

Variables		Intervention N=198
Used any of the POP that the pharmacist gave	Yes	158/198(79.8%)
	No	35/198(17.7%)
	Missing	5/198(2.5%)
If not, why*	Not with a regular partner	8/35(22.9%)
	Not requiring regular contraception	7/35(20.0%)
	Worried about possible side effects	10/35(28.6%)
	Didn't understand to use it	1/35(2.9%)
	Preferred to start another contraceptive	6/35(17.1%)
	Used POP in the past and it did not agree	1/35(2.9%)
	Preferred to see GP for contraception	2/35(5.7%)
	Other	10/35(28.6%)
When did the participant start the POP	Same day took EC	27/158(17.1%)
	Day after took EC	93/158(58.9%)
	With the start of next period after EC	19/158(12.0%)
	Other	16/158(10.1%)
	Missing	3/158(1.9%)
Number of packets of POP used	< 1 packet	15/158(9.5%)
	1 packet	17/158(10.8%)
	< 2 packets	13/158(8.2%)
	2 packets	8/158(5.1%)
	< 3 packets	10/158(6.3%)
	3 packets	70/158(44.3%)
	Still taking the POP	22/158(13.9%)
	Missing	3/158(1.9%)
Main reason for stopping POP before the supply ran out	Due to side effects	40/158(25.3%)
	Started another method	6/158(3.8%)
	Other	22/158(13.9%)
	Missing	90/158(57.0%)

EC= emergency contraception

*More than one answer could be given

Supplemental Table 8: Use of rapid access card to get appointment at the Sexual and Reproductive Health (SRH) Clinic

Variables		Intervention	
		Period 1	Period 2
		N=147	N=51
Did the pharmacist give the participant a 'rapid access card' to get an appointment at the study SRH clinic	Yes	105/147(71.4%)	32/51(62.7%)
	No	25/147(17.0%)	5/51(9.8%)
	I cannot remember	12/147(8.2%)	12/51(23.5%)
	Missing	5/147(3.4%)	2/51(3.9%)
Did the participant attend the study SRH clinic	Yes	25/117(21.4%)	6/44(13.6%)
	No	90/117(76.9%)	35/44(79.5%)
	Missing	2/117(1.7%)	3/44(6.8%)
If no, why	Not requiring contraception	19/90(21.1%)	9/35(25.7%)
	Preferred to see GP for contraception	45/90(50.0%)	17/35(48.6%)
	Preferred to attend another SRH clinic for contraception	5/90(5.6%)	0
	Other	22/90(24.4%)	8/35(22.9%)
When did the participant go to the SRH clinic	The same day took the EC	1/25(4.0%)	0
	< 1 month after the EC	1/25(4.0%)	0
	1 to 2 months after the EC	2/25(8.0%)	1/6(16.7%)
	2 to 3 months after the EC	12/25(48.0%)	3/6(50.0%)
	3 to 4 months after the EC	4/25(16.0%)	1/6(16.7%)
	Other	4/25(16.0%)	0
	Missing	1/25(4.0%)	1/6(16.7%)
Did participant remember to take rapid access card to get appointment at study SRH clinic	Yes	14/25(56.0%)	3/6(50.0%)
	No	11/25(44.0%)	2/6(33.3%)
	Missing	0	1/6(16.7%)
If participant did not take rapid access card, were they refused an appointment	Yes	1/11(9.1%)	0
	No	10/11(90.9%)	2/2(100.0%)
How long did participant wait to be seen at the SRH clinic	< 30 mins	8/25(32.0%)	3/6(50.0%)
	< 1 hour	7/25(28.0%)	1/6(16.7%)
	1-2 hours	8/25(32.0%)	1/6(16.7%)
	Other	2/25(8.0%)	0
	Missing	0	1/6(16.7%)
Did study SRH clinic provide participant with a method of contraception	Yes	19/25(76.0%)	5/6(83.3%)
	No	6/25(24.0%)	0
	Missing	0	1/6(16.7%)
Did SRH clinic provide participant with the participants preferred method of contraception	Yes	16/25(64.0%)	5/6(83.3%)
	No	8/25(32.0%)	0
	Missing	1/25(4.0%)	1/6(16.7%)
If no, why	Not enough staff or time	2/8(25.0%)	0
	Risk of pregnancy	1/8(12.5%)	0
	Other	6/8(75.0%)	0
Method of contraception received	Progestogen only implant	1/25(4.0%)	0
	Progestogen only injectable	0	0
	Cu-IUD	1/25(4.0%)	0
	LNG-IUS	2/25(8.0%)	1/6(16.7%)
	Combined hormonal contraceptive pill/patch/ring	3/25(12.0%)	0
	Progestogen only pill	12/25(48.0%)	4/6(66.7%)
	Male condom	2/25(8.0%)	0
Experience of the rapid access system to study SRH clinic	Smooth	16/25(64.0%)	3/6(50.0%)
	Neither/Nor	6/25(24.0%)	2/6(33.3%)
	Problematic	1/25(4.0%)	0
	Missing	2/25(8.0%)	1/6(16.7%)

Supplemental Table 9. Data from the study Sexual and Reproductive Health (SRH) clinics.

		Intervention	Control
		N=305	N=309
Attended the study SRH clinic	No	253/305(83.0%)	266/309(86.1%)
	Yes	52/305(17.0%)	43/309(13.9%)
Method of contraception provided at this visit	Yes	26/52(50.0%)	15/43(34.9%)
Progestogen only implant		2/26(7.7%)	3/15(20.0%)
Cu-IUD		1/26(3.8%)	4/15(26.7%)
LNG-IUS		1/26(3.8%)	3/15(20.0%)
Progestogen only injectable		0	0
Combined hormonal contraceptive (pill/patch/ring)		3/26(11.5%)	1/15(6.7%)
POP		16/26(61.5%)	2/15(13.3%)
Male Condom		3/26(11.5%)	3/15(20.0%)
Other Method		1/26(3.8%)	0

POP= progestogen only pill
Cu-IUD = copper bearing intrauterine device
LNG-IUS= levonorgestrel releasing intrauterine system
